# Evaluation of Electromechanical Properties and Conversion Efficiency of Piezoelectric Nanocomposites with Carbon-Fiber-Reinforced Polymer Electrodes for Stress Sensing and Energy Harvesting

**DOI:** 10.3390/polym13183184

**Published:** 2021-09-19

**Authors:** Yaonan Yu, Fumio Narita

**Affiliations:** Department of Frontier Sciences for Advanced Environment, Graduate School of Environmental Studies, Tohoku University, Sendai 980-8579, Japan; yu.yaonan.p5@dc.tohoku.ac.jp

**Keywords:** piezoelectricity, laminated beam theory, bending test, piezoelectric nanocomposites, stress sensor, energy harvester

## Abstract

Wireless sensor networks are the future development direction for realizing an Internet of Things society and have been applied in bridges, buildings, spacecraft, and other areas. Nevertheless, with application expansion, the requirements for material performance also increase. Although the development of carbon-fiber-reinforced polymer (CFRP) to achieve these functions is challenging, it has attracted attention because of its excellent performance. This study combined the CFRP electrode with epoxy resin containing potassium sodium niobate piezoelectric nanoparticles and successfully polarized the composite sample. Furthermore, a three-point bending method was applied to compare the bending behavior of the samples. The peak output voltage produced by the maximum bending stress of 98.4 MPa was estimated to be 0.51 mV. Additionally, a conversion efficiency of 0.01546% was obtained. The results showed that the piezoelectric resin with CFRPs as the electrode exhibited stress self-inductance characteristics. This study is expected to be applied in manufacturing self-sensing piezoelectric resin/CFRP composite materials, paving the way for developing stable and efficient self-sensing structures and applications.

## 1. Introduction

The Internet of Things (IoT) links everything to the Internet and provides significant innovation for society. Sensors and power supplies will be installed in various items such as clothes, equipment, and factories. Data, such as real-time conditions, are accumulated on the ground via the Internet, analyzed, and fed back to the real world [1]. The number of sensors and batteries necessary to realize the IoT society is expected to reach one trillion by 2030. Preparing so many sensors and batteries poses several societal issues such as high cost, an almost impossible number of cable connections, and battery replacement [2].

The piezoelectric effect is a phenomenon in which a voltage is generated on the basis of the strain produced when mechanical loads are applied to quartz or specific types of ceramics. The piezoelectric effect has been used to develop various sensors [3,4,5,6,7,8]. Hence, it has recently emerged as a potential material for vibration energy harvesting that can recover electric power from unused energy such as vibration [9,10,11,12,13,14,15,16,17].

Lead zirconate titanate (PZT) is a type of piezoelectric ceramic and crystal group with a perovskite structure. PZT has a high dielectric constant, piezoelectricity, and ferroelectricity, among piezoelectric ceramics, and its raw material is relatively inexpensive, allowing mass production. It has good sensitivity and a significant electromechanical coupling coefficient, since it is relatively easy to induce polarization. However, PZT has the drawback of brittleness, which makes it less durable [18,19]. Additionally, lead, which is a harmful substance, is applied. Thus, the development of piezoelectric composite materials in which lead-free piezoelectric particles are dispersed in resin is receiving attention [20,21].

Fiber-reinforced polymers (FRPs) are composite materials composed of lightweight, high-strength polymers and different fibers [22,23]. Polymer is not suitable as a structural material by itself, because it is lightweight and has a low elastic modulus. Thus, high-strength FRP was developed by combining polymers with various fibers such as glass or carbon fiber. FRP is inexpensive, lightweight, and durable and has been used in various components such as the hull of small vessels, the interior and exterior of aircraft, automobiles and railroad vehicles, and housing equipment such as unit baths and septic tanks. Particularly, carbon-fiber-reinforced polymer (CFRP) is increasingly being used in structural parts of automobiles and aircraft to minimize fuel consumption and weight [24,25,26,27,28,29]. Adding a piezoelectric effect to this CFRP not only allows automobiles and aircraft to have a stress sensor function but also allows for extracting electrical energy from their vibrations. Narita et al. [30] recently fabricated a potassium sodium niobate (KNN) nanoparticle-filled epoxy interlayer with copper electrodes sandwiched between two CFRP laminates. Additionally, they demonstrated that more than 50 mV output voltage can be generated from the impact load. Although it is convenient to laminate CFRPs to piezoelectric materials using CFRP prepregs, the depolarization during the curing process significantly affects the piezoelectric properties. Alternatively, interply adhesion should be considered. Nevertheless, the problem of interply adhesion between various materials is complicated. Using CFRPs as electrodes will directly result in a low-cost, lightweight, and simple construction. Wang et al. [31] investigated the piezoelectric coefficient, *d*_33_, of KNN piezoelectric resin with CFRP electrodes, successfully fabricated CFRP energy harvesting structures, and evaluated the output power by impact load and bending vibration. The results showed that approximately 0.8 μW/cm^3^ of power was generated from impact tests and approximately 4 μW/cm^3^ of power was obtained from bending vibration tests. However, the epoxy resin in CFRP is an insulating material, which decreases the piezoelectric properties of piezoelectric/CFRP composite materials. Thus, the conductivity of the CFRP electrode must be improved, resulting in piezoelectric/CFRP composite materials with excellent mechanical properties while maintaining piezoelectric properties. This is a significant advantage for multifunctional structural composite materials.

With the rapid development of the world’s transportation infrastructure, some realistic applications of piezoelectric materials for smart sensing have been undertaken. Piezoelectric sensors are widely used, as structural health monitoring (SHM) [32] and electro-mechanical (EM) preload monitoring is essential to ensure the safety of bridges during their service life [33].

This study combined CFRP electrodes with epoxy resin containing KNN piezoelectric nanoparticles, which is a continuation of previous work [31]. The characteristics of the output voltage were evaluated under a three-point bending test to demonstrate that CFRPs can be used as an electrode. Additionally, the conversion rate of the piezoelectric composite material was calculated through its bending behavior. The results revealed that CFRP can be directly used as an electrode, and the piezoelectric composite material can be applied to stress self-sensor and energy harvester.

## 2. Experimental Procedure

**Figure 1:**
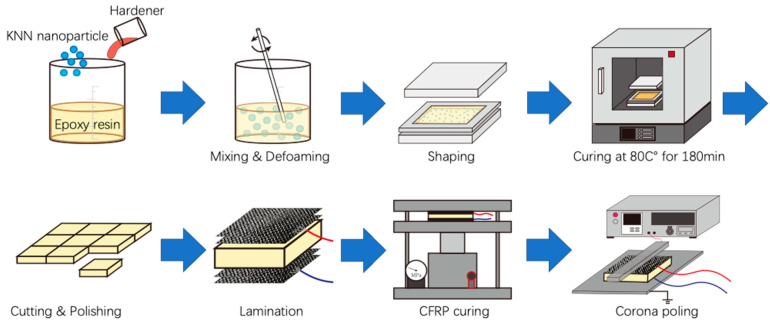
Schematic for the complete fabrication process of piezoelectric composite specimens.

Figure 1 shows the fabrication process of carbon-fiber-reinforced piezoelectric nanoparticle-dispersed epoxy resin. KNN nanoparticles (Nippon Chemical Industrial Co., Ltd., Tokyo, Japan) were used as piezoelectric fillers. KNN nanoparticles were mixed with bisphenol-F epoxy resin (Daido Co., Ltd., Tokyo, Japan) for 30 min using a planetary mixer and defoamed for 10 min, after which the hardener (Mitsubishi Chemical Co., Ltd., Tokyo, Japan) was added and remixed for 10 min and defoamed for 5 min. The volume fraction of KNN nanoparticles was maintained at 30 vol.%. The mixture was spread on a mold and cured at 80 °C for 3 h to control the specimen’s dimensions. After curing, the specimen was cut and polished to 40 mm × 6 mm.

CFRP prepregs (F6347B-05P #2500, TORAY INDUSTRIES, INC., Tokyo, Japan) were introduced as the reinforcement material and electrode of piezoelectric epoxy resin. Figure 1 shows that the CFRPs were compounded on the upper and lower parts of the KNN/EP composites, whereas the wires were buried between two layers of CFRP prepregs. After lamination, the prepregs were cured at 130 °C and 0.5 MPa for 60 min. Excess CFRPs were removed and polished after curing.

**Figure 2:**
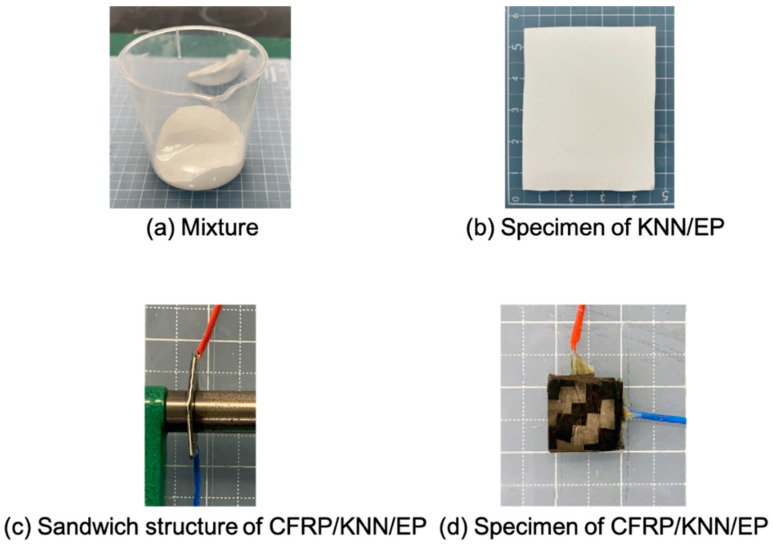
Images during fabrication of the specimens: (**a**) the piezoelectric nanoparticle and epoxy resin with hardener was entirely mixed; (**b**) the KNN/EP specimen was homogeneous, and the molding quality was relatively good. Before spreading the mixture on the mold, it was necessary to coat the mold with release wax; (**c**) the sandwich structure of the CFRP/KNN/EP composite material; (**d**) wires embedded in the upper and lower CFRP electrodes.

Notably, some processes are recorded in Figure 2. Figure 2a shows the mixture after mixing. First, the KNN nanoparticle and epoxy resin are completely mixed without the hardener, since the hardener will rapidly increase the viscosity of the resin solution, which will inhibit nanoparticle dispersion in the composites. After mixing, defoaming is required, which can effectively enhance molding quality. The sample in Figure 2b comprised a homogeneous material with high molding quality. It was necessary to coat the mold with release wax before spreading the mixture on it. This is because the piezoelectric epoxy resin is brittle and is easily damaged during the release. The mold–release wax can also guarantee the quality of the piezoelectric epoxy resin. Figure 2c,d show the piezoelectric composite (CFRP/KNN/EP) sandwich structure. Wires were connected to the upper and lower CFRPs. Because carbon fibers exhibit conductivity, CFRPs may be used as an electrode and structural reinforcement. In the CFRP molding process, the upper and lower electrodes were easily short-circuited because of the flowing resin under heat and pressure. Thus, excess CFRPs must be removed after the CFRPs are cured. The sample was then subjected to corona poling treatment to stimulate the piezoelectricity after completing the aforementioned steps.

**Figure 3:**
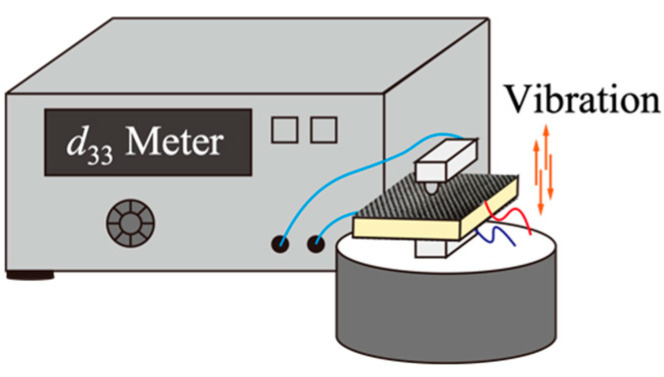
The *d*_33_ measurement equipment.

The specimen was polarized in the thickness direction using a corona polarization device (ELC-01 N, Element Co., Ltd., Kawasaki, Japan) at 75 °C and a 16 kV/mm electric field for 30 min. The electric properties of the material were measured as shown in Figure 3, and the longitudinal direct piezoelectric coefficient, *d*_33_, was determined.

**Figure 4:**
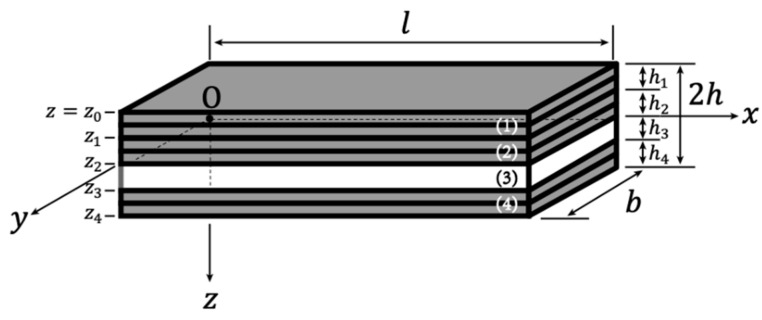
Schematic of the structure of KNN/EP with CFRP electrodes.

We propose the design of an asymmetric stress self-sensor in this experiment. This design was proposed because the neutral axis (NA) of the sensor is a region of zero stress when it is under bending. Moving the piezoelectric material layer out of the NA benefits the piezoelectric material by allowing it to receive more stress and improving the sensor’s sensitivity. Figure 4 shows the laminated structure of the specimen. In this study, a double-layer was considered one layer for simplicity. The upper electrode was a four-layer structure CFRP, and the lower electrode was a two-layer CFRP. From Figure 4, $h_{3}$ is the thickness of the piezoelectric KNN/EP material and was approximately 0.3 mm; $h_{4}$ shows that the thickness of a two-layer CFRP was the same as $h_{3}$, and the thickness of the upper electrode, $h_{1}+h_{2}$, was equal to that of the lower electrode, $h_{3}+h_{4}$. The total thickness of specimen 2h was approximately 1.42 mm.

**Figure 5:**
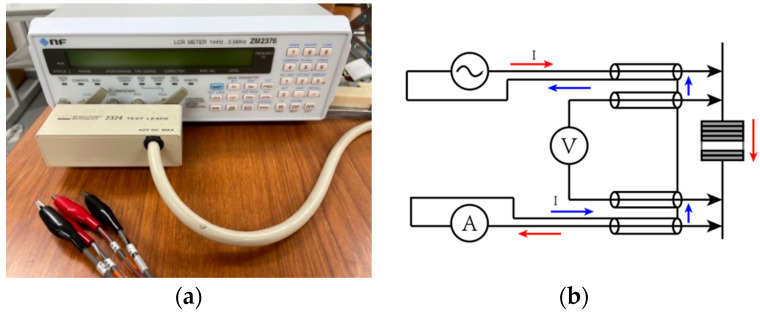
(**a**) LCR meter (ZM2376, NF Co., Ltd., Japan); (**b**) four-terminal connection method for the LCR meter system.

An LCR meter (ZM2376, NF Co., Ltd., Yokohama, Japan) is a device used to measure the capacitance ($C_{p}$) of an electronic component (Figure 5a). The four-terminal Kelvin connections were used in this study to measure the specimen’s permittivity at a voltage of 1 V at a frequency of 1 kHz. With two terminal pairs available, one can measure the voltage across the specimen with one pair and apply a current to the specimen with the other pair (Figure 5b). The Kelvin connection minimizes errors due to the cabling and connection to the specimen. The capacitance of the specimen obtained from the measurement was approximately 56.97 pF. Hence, the sample’s relative permittivity,$\;\epsilon_{r}$, was calculated from the following equation:

(1) $$\epsilon_{r}=\frac{C_{p}\cdot h_{3}}{\epsilon_{0}\cdot S}\;$$

where $\epsilon_{0}$ is the vacuum permittivity ($8.854\;\mathrm{pF}\cdot m^{-1}$), and the area, *S*, of the electrodes was approximately 228${\;\mathrm{mm}}^{2}$. Therefore, the relative permittivity,$\;\epsilon_{r}$, of the KNN/EP material with CFRP electrodes was 40.07.

## 3. Analytical Procedure

**Figure 6:**
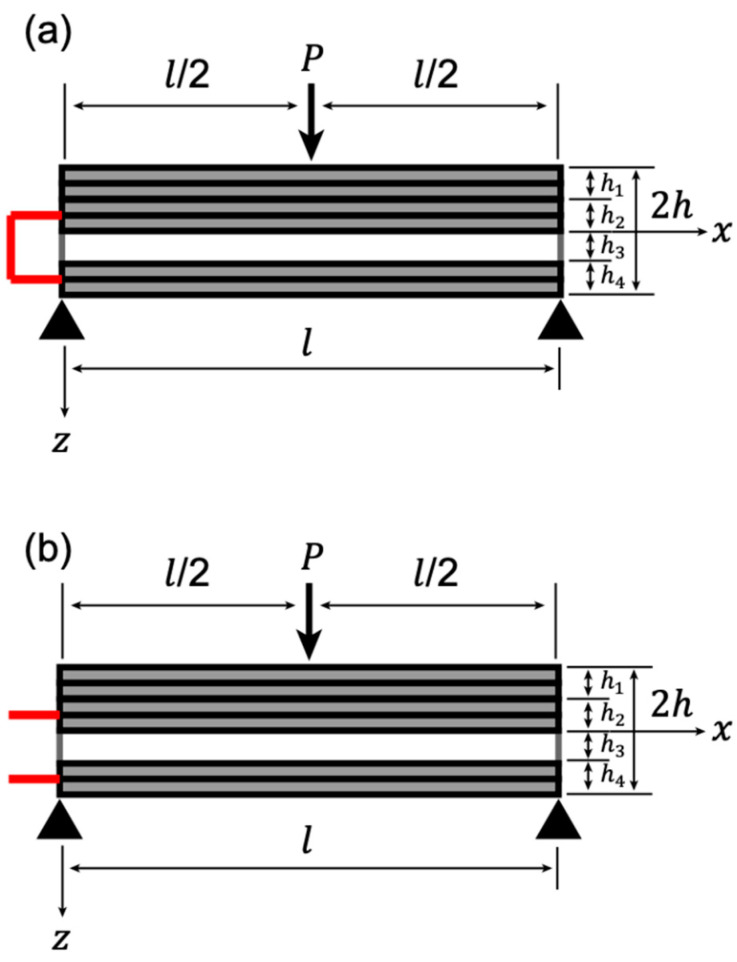
Schematic of a centrally loaded composite beam for three-point bending test: (**a**) short circuit condition; (**b**) open circuit condition.

We considered a four-layer composite beam of length, l (32 mm), width b (5.8 mm), and total thickness of 2h (1.42 mm) under three-point bending (Figure 6). Let the coordinates on the x- and y-axis be chosen such that they coincide with the composite beam’s middle plane and the *z*-axis perpendicular to this plane. The CFRP electrode layers were added to the upper and lower surfaces of a piezoelectric nanoparticle-dispersed epoxy resin poled in the *z*-direction. The *k*th layer had a thickness, $h_{k}=z_{k}-z_{k-1}\;\left(k=1,2,3,4\right)$, where $z_{0}=-h$ and $z_{4}=h$.

The layers were free to expand vertically in the composite beam with a concentrated load *P* at the center (*x* = *l*/2), implying that the stress, ${\left(\sigma_{zz}\right)}_{k}$, was equal to 0. Additionally, we may assume that the stress ${\left(\sigma_{yy}\right)}_{k}\;$ was 0 because the composite beams were considered long and slender. No shear stresses will develop since the polarization is not perpendicular to the electric field ${\left(E_{z}\right)}_{k}$, and we may infer that no shear strains exist, reducing the nontrivial stresses to ${\left(\sigma_{xx}\right)}_{k}$. Hence, the constitutive equation for the piezoelectric resin (third layer) concerning the reference axes of the composite beam (x, z) can be written as follows:

(2) $${\left(\varepsilon_{xx}\right)}_{3}={\left(s_{11}\right)}_{3}{\left(\sigma_{xx}\right)}_{3}+{\left(d_{31}\right)}_{3}{\left(E_{z}\right)}_{3}$$

(3) $${\left(D_{z}\right)}_{3}={\left(d_{31}\right)}_{3}{\left(\sigma_{xx}\right)}_{3}+{\left(\epsilon_{33}\right)}_{3}{\left(E_{z}\right)}_{3}$$

where $\varepsilon_{xx}$ is the strain, $D_{z}$ is the electric displacement, ${\left(s_{11}\right)}_{3}=s_{11}$ is the elastic compliance, ${\left(d_{31}\right)}_{3}=d_{31}$ is the transverse direct piezoelectric coefficient, and ${\left(\epsilon_{33}\right)}_{3}=\epsilon_{33}$ is the permittivity. For a short circuit condition (Figure 6a), the electric field within the piezoelectric resin is 0, i.e., ${\left(E_{z}\right)}_{3}=0$. Therefore, Equation (2) becomes the following:

(4) $${\left(\varepsilon_{xx}\right)}_{3}={\left(s_{11}\right)}_{3}{\left(\sigma_{xx}\right)}_{3}$$

where

(5) $${\left(s_{11}\right)}_{3}=s_{11}=\frac{1}{E_{11}^{\mathrm{piezo}}}\;$$

and $E_{11}^{\mathrm{piezo}}$ is Young’s modulus of the piezoelectric KNN/EP material in the *x*-direction. For an open circuit condition (Figure 6b), the electric displacement in the piezoelectric resin is 0, i.e., ${\left(D_{z}\right)}_{3}=0$. Furthermore, the electric field within the piezoelectric resin due to the mechanical loading is obtained as follows:

(6) $${\left(E_{z}\right)}_{3}=-\frac{d_{31}}{\epsilon_{33}}{\left(\sigma_{xx}\right)}_{3}$$

Substituting Equation (6) into Equation (2) results in the following:

(7) $${\left(\varepsilon_{xx}\right)}_{3}=\left(s_{11}-\frac{d_{31}{}^{2}}{\epsilon_{33}}\right){\left(\sigma_{xx}\;\right)}_{3.}$$

The CFRPs (first, second, and fourth layers) constitutive equation can be given as follows:

(8) $${\left(\varepsilon_{xx}\right)}_{k}={\left(s_{11}\right)}_{k}{\left(\sigma_{xx}\right)}_{k\;\;}\left(k=1,\;2,\;4\right)$$

where

(9) $${\left(s_{11}\right)}_{k}=\frac{1}{E_{11}^{\mathrm{cf}}}\;\;\;\left(k=1,\;2,\;4\right),\;\;$$

and $E_{11}^{\mathrm{cf}}$ is CFRP Young’s modulus in the *x*-direction. 

The moment, M, in the composite beam (Figure 6) can be obtained as follows:

(10) $$M=\frac{Px}{2}\;\left(0\leq x\leq \frac{l}{2}\right).\;\;\;$$

The differential equation for the displacement, w, is obtained as follows:

(11) $$\frac{d^{2}w}{dx^{2}}=-\frac{Px}{2D}\;\left(0\leq x\leq \frac{l}{2}\right),\;$$

where D is the bending modulus of the composite beam and can be expressed as follows:

(12) $$D=b\overset{4}{\underset{k=1}{\sum }}\int_{z_{k-1}}^{z_{k}}\frac{1}{{\left(s_{11}\right)}_{k}}z^{2}dz\;\;\;=\frac{bh^{3}}{3}\;\left(\frac{15}{8}E_{11}^{\mathrm{cf}}+\frac{1}{8}E_{11}^{\mathrm{piezo}}\right).\;\;\;\;\;\;\;\;\;\;\;$$

The beam is subjected to the following boundary conditions:

(13) $$w=0\;\left(x=0\right),\;\;\frac{dw}{dx}=0\;\left(x=\frac{l}{2}\right)$$

The load-point (x=l/2) displacement is given by the following:

(14) $$w=\frac{Pl^{3}}{48D}\;\;=\;\;\frac{Pl^{3}}{32E_{11}^{c}bh^{3}},\;\;\;$$

where $E_{11}^{c}=\left(15E_{11}^{\mathrm{cf}}+E_{11}^{\mathrm{piezo}}\right)/16$ is Young’s modulus of the CFRP/KNN/EP composite material and superscript “c” represents the composite. The upper surface of the bending beam is in compression, and the bottom surface is in tension for the simply supported structural beam. Thus, the maximum tensile stress farthest from the central axis can be obtained when z=h, i.e.,

(15) $$\sigma_{\max}=\frac{3Pl}{8bh^{3}}.\;$$

## 4. Results and Discussion

### 4.1. Material Properties

**Figure 7:**
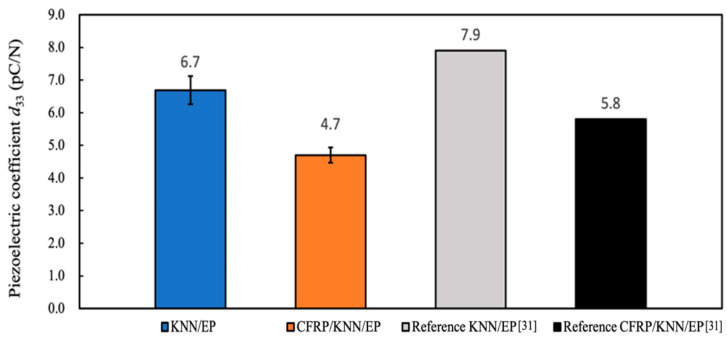
Comparison of the piezoelectric coefficients, *d*_33_.

The piezoelectric coefficient, *d*_33_, of the KNN/EP and CFRP/KNN/EP materials was tested and compared with the reference [31]. Figure 7 shows that the piezoelectric coefficients of the specimens generated in this experiment were lower than those of the previous samples [31]. The piezoelectric coefficients of the composites were significantly reduced, especially after combining with CFRPs.

The polarization was made in the thickness direction in this study. Hence, *d*_33_ represents the coefficient of charge per unit force in the polarization direction. The *d*_33_ meter system can detect the piezoelectric element’s mechanical strain per unit of applied electrical energy or the electrical energy generated by the element per unit of applied mechanical stress. A low-frequency force is applied in the thickness direction when using a *d*_33_ meter system, and the charge changes are detected. CFRP was used as the electrode in this experiment instead of metal. Although carbon fiber is a good conductor, the polymer in carbon fiber composite materials is insulated, thereby decreasing the result of measuring the *d*_33_ coefficient. 

Thus, it is necessary to consider whether carbon fibers are in good contact when CFRPs are used as an electrode. The carbon fiber fabric inside the CFRP electrode adopted a twill weave in this study. The carbon fibers inside the composite had more contact points than the one-directional carbon-fiber composite material ($<0.25\;\Omega /{\mathrm{mm}}^{2}$), which helps improve conductivity. However, the measured resistance of the CFRP electrode was approximately $1.25\;\Omega /{\mathrm{mm}}^{2}$. Additionally, the electrodes were combined with the piezoelectric material via the conductive epoxy resin once the surface was polished.

**Figure 8:**
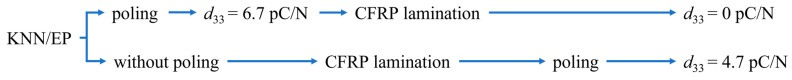
Processes and results of the two fabrication methods.

The piezoelectric coefficient depends on the matrix material and piezoelectric particles. Nevertheless, the findings revealed that the combination with CFRPs caused the piezoelectric coefficient to decrease. Moreover, we discovered that the KNN/EP material lost its piezoelectricity after being compounded with CFRPs (Figure 8). Hence, we adjusted the polarization step to after the CFRP lamination, and Figure 8 shows the results. The *d*_33_ of the CFRP/KNN/EP piezoelectric composite could reach approximately 4.7 pC/N, indicating that the piezoelectric polymer can be effectively polarized when CFRPs were used as the electrode.

**Figure 9:**
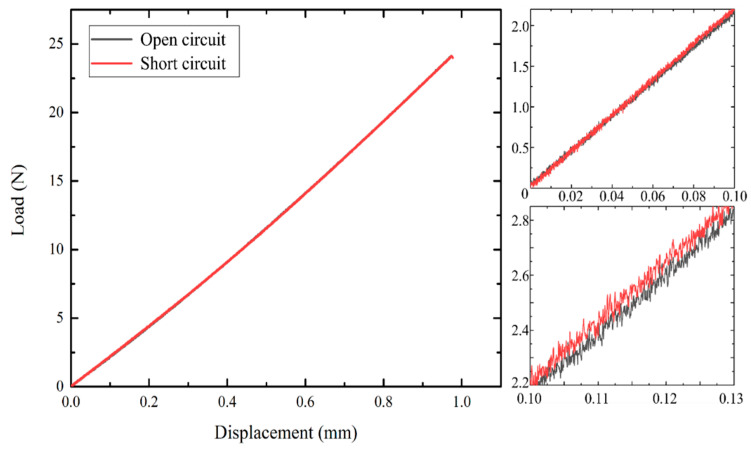
Load–displacement curve of the open circuit and short circuit situations.

Figure 9 shows the three-point bending test results for the open circuit and short circuit situations. The three-point bending test was based on Japanese Industrial Standards (JIS) K 7074 [34]; the specimen was the 2/5 size of JIS K 7074, except for the thickness. The three-point bending test was performed using a universal testing machine (Autograph AG-50kNXD, Shimadzu Corporation, Kyoto, Japan) at a speed of 1 mm/min. A cyclic bending mode was used to ensure data stability. The maximum displacement was limited to 1 mm, and the sample was circled five times within the 0.5–1 mm range. Each sample was tested at least four times, and the average value was recorded. The load–displacement curve was observed to be different because of the influence of transverse piezoelectricity on the material, and this influence corresponds to Equations (4) and (7). According to the load–deflection diagram, Young’s modulus,$\;E_{11}^{c}$, of the CFRP/KNN/EP composite material can be calculated from Equation (14). Therefore, Young’s modulus, ${\left(E_{11}^{c}\right)}_{\mathrm{short}}$, in the short circuit condition is approximately 13.1 GPa; furthermore, the elastic compliance in the short circuit $\mathrm{condition}\;{\left(s_{11}^{c}\right)}_{\mathrm{short}}\;$ can be obtained by first substituting the value of ${\left(E_{11}^{c}\right)}_{\mathrm{short}}\;$ in Equation (7). Moreover, the calculated Young’s modulus, ${\left(E_{11}^{c}\right)}_{\mathrm{open}}$, in the open circuit condition was approximately 12.2 GPa, and the reciprocal of this result was ${\left(s_{11}^{c}\right)}_{\mathrm{short}}-d_{31}{}^{2}/\epsilon_{33}$. Hence, if we measure Young’s modulus, $E_{11}^{\mathrm{piezo}}$, of the KNN/EP material, we can predict the *d*_31_ coefficient.

### 4.2. Electrical Energy

**Figure 10:**
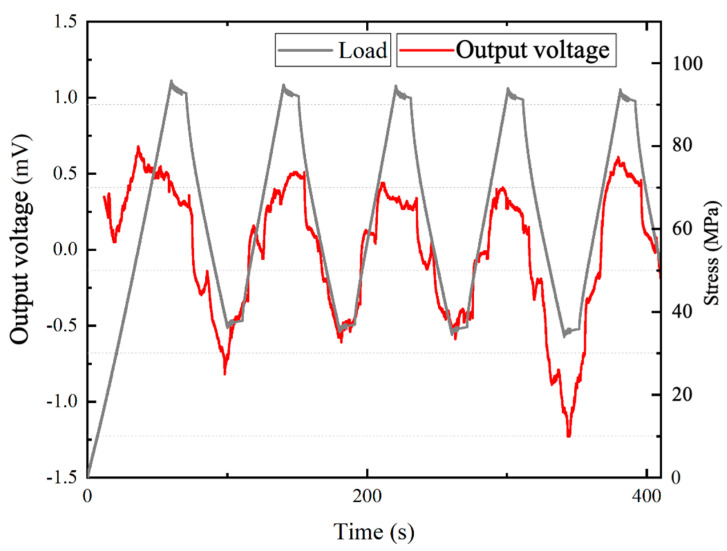
Three-point bending test: measured peak-to-peak output voltage.

To evaluate the electrical energy generated by the CFRP/KNN/EP composite material’s cyclic bending, the peak-to-peak output voltage, $V_{\mathrm{pp}}$, was recorded using a data logger (Keyence NR-500, Keyence Co., Osaka, Japan) in the three-point bending test. To apply a load to the CFRP/KNN/EP composite material, the sample was first fixed on the fulcrum, and then the maximum bending displacement was set to 1 mm with a test speed of 1 mm/s. The experimental results are shown in Figure 10. Although the output voltage was unstable at the beginning of the load stage, the relationship between the output voltage and the bending stress was still obvious. 

The output electric energy,$\;U_{e}$, was evaluated on the basis of the load resistance, R (approximately 300 Ω), the measured output voltage, $V_{\mathrm{out}}$, and the time, t. Therefore, the output electric energy is given as follows:

(16) $$U_{e}=\int^{\;}\frac{V_{\mathrm{out}}{\left(t\right)}^{2}}{R}dt$$

**Figure 11:**
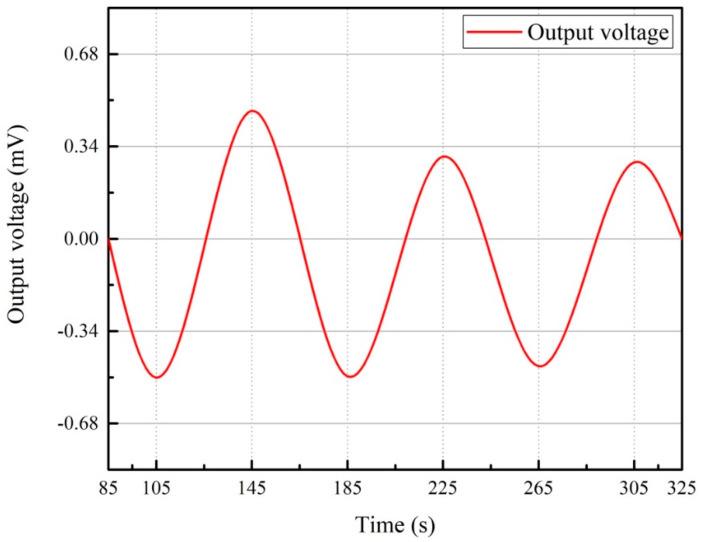
Relationship between the output voltage and time in cyclic bending.

Because the bending stress clearly correlates with the output voltage, the curve was fitted by taking the extreme value. We fit the output voltage value to a curve, which could facilitate the observation of the change of the output voltage during the bending test and predict the future trend. The preset displacement was reached after 85 s. The average output peak voltage ${\bar{|V}}_{\mathrm{pp}}|$ = 0.51 mV was measured during cyclic bending with 80 s per cycle. Additionally, since the piezoelectric material generates an alternating voltage (Figure 11), the relationship between voltage and time can be written as follows:

(17) $$V_{\mathrm{out}}=-{\bar{V}}_{\mathrm{pp}}\sin\frac{\pi }{40}t.$$

As an example, electric energy output ($\;U_{e}$) can be calculated using Equation (16), where *t* = 145 s is the time to initially reach the peak. Therefore, the actual output power in the three-point bending test was $6.67\times 10^{-6}\;J$.

### 4.3. Strain Energy

Furthermore, the strain energy in the composite beam can be written as follows:

(18) $$U_{s}=\frac{2}{2{\left(E_{11}^{c}\right)}_{o}I}\overset{l/2}{\underset{0}{\int }}{\left(M\right)}^{2}dx=\frac{P^{2}l^{3}}{24{\left(E_{11}^{c}\right)}_{o}I},$$

where $I=b{\left(2h\right)}^{3}/12$ is the moment of inertia of the rectangle cross-section. ${\left(E_{11}^{c}\right)}_{\mathrm{open}}=12.2\;\mathrm{GPa}\;$ was evaluated from the bending test in the previous section. Furthermore, the output voltage reached the peak value and the applied load, P, was 23.1 N. Thus, $U_{s}=0.04315\;J$. The total energy conversion efficiency, $\left(\eta_{\mathrm{piezo}}/\%\right)$, was calculated on the basis of the ratio of the output electrical energy ($6.67\times 10^{-6}\;J$) to the input mechanical energy, $U_{s}$. We determined the conversion efficiency to be $\eta_{\mathrm{piezo}\;}$ = 0.01546%.

## 5. Conclusions

In this study, we proposed a design for an asymmetric stress self-sensor using potassium sodium niobate nanocomposites with CFRP electrodes. The piezoelectric material can be successfully polarized when the CFRPs are used as an electrode by optimizing the test procedure and adjusting the polarization after the CFRP laminate. The three-point bending test demonstrated that the transverse piezoelectric coefficient can be calculated using bending behavior, and the output voltage generated by the test was stable. Additionally, the piezoelectric efficiency can be calculated from the conversion of electrical and strain energies, which offers the feasibility of practicability.

In conclusion, the optimization of hybrid CFRP composite materials will result in the progress and development of stress self-sensing or energy harvesting in the future. Furthermore, the maximum force limit of piezoelectric polymers has increased since CFRP composites have high mechanical properties and lightweight characteristics. It is a promising structural reinforcing material with development potential in the field of structural health sensors for bridges and spacecraft.
